# Infrared Thermography and Machine Learning for Mastitis Detection in Dairy Cows: A Pilot Case Study in Egyptian Farms

**DOI:** 10.3390/vetsci13070640

**Published:** 2026-06-30

**Authors:** Aya S. Elmasry, Eman A. Elwakeel, Ali M. Allam, Marwa F. A. Attia, Alaa. T. Elmaria, Elsayed. E. M. Badr, Sobhy M. A. Sallam

**Affiliations:** 1 Department of Animal and Fish Production, Faculty of Agriculture, Alexandria University, Alexandria 21545, Egypt; ayaabdelkader2020@alexu.edu.eg (A.S.E.); emankeel@alexu.edu.eg (E.A.E.); ali.alam@alexu.edu.eg (A.M.A.); 2 Animal Production Research Institute, Agricultural Research Center, Giza 12618, Egypt; marwa.elfeky@alexu.edu.eg; 3 Department of Artificial Intelligence, Faculty of Engineering, Mansoura National University, Gamasa 33515, Egypt; alaatahamohamed@mansnu.edu.eg; 4 Department of Scientific computing, Faculty of Computers and Artificial Intelligence, Benha University, Benha 19111, Egypt; alsayed.badr@fci.bu.edu.eg; 5 Department of Animal and Poultry Production, College of Agriculture and Food, Qassim University, Buraydah 51452, Saudi Arabia

**Keywords:** artificial intelligence, livestock, machine learning, mastitis, smart farming

## Abstract

Mastitis is a major and costly dairy disease that reduces milk yield and quality and harms animal welfare. This study evaluated infrared thermography (IRT) combined with machine learning (ML) for non-invasive mastitis screening in dairy cows and explored links with biological and feeding-system variables in Egyptian farms. A total of 976 thermal udder images obtained from 488 Holstein cows were used, including 708 healthy and 268 mastitic images. Images were captured before milking, processed with CLAHE, resized to 224 × 224 pixels, and split using cow-level grouping before augmentation to prevent animal-level data leakage. The training set contained 780 original images and was augmented to a balanced 4708-image set (2354 per class), while the held-out test set remained unaugmented, with 196 original images (142 healthy and 54 mastitic). EfficientNetB3 with global average and max pooling extracted 3072 thermal features, and ten ML classifiers were evaluated. In the image-level hold-out evaluation, MLP achieved the best performance (accuracy = 86.22%, AUC = 0.9184, sensitivity = 74.07%, specificity = 90.85%), followed by SVM (accuracy = 83.67%, AUC = 0.8963). A separate group-based five-fold cross-validation yielded a more conservative AUC of 0.6812 ± 0.1323 and accuracy of 0.6244 ± 0.0642. Logistic regression analyses did not identify statistically significant associations between model predictions and somatic cell count (SCC), California Mastitis Test (CMT), blood biomarkers, or nutritional variables at *p* < 0.05. Ration A (Delta Misr) showed a higher observed mastitis incidence (20/40; 50.0%) than Ration B (Copenhagen; 16/45; 35.6%), but nutritional predictors were not statistically significant, indicating that farm-level confounding should be considered. Overall, IRT with ML remains a promising non-invasive screening approach, but broader multicenter datasets and independent external validation are needed before routine farm deployment.

## Simple Summary

Mastitis is one of the most common and economically important diseases in dairy cattle because it affects animal health, welfare, milk yield, and milk quality. This pilot study examined whether infrared thermal images of the udder, combined with machine-learning classifiers, could support mastitis screening in Holstein dairy cows. Several classifiers were tested, and the multi-layer perceptron and support vector machine models showed the best image-level performance. This study also explored selected biological and feeding-related variables, but these findings should be interpreted cautiously because they were exploratory. Overall, the results suggest that infrared thermography combined with machine learning may be useful as a supportive, non-invasive screening approach, although larger independent studies are still needed before routine farm application.

## 1. Introduction

The animal production sector faces numerous challenges, including scarcity and fluctuating year-round feed supplies, as well as high feed prices, climatic changes, heat stress, water and land use issues, supply chain disruptions, health risks, and zoonotic diseases. Mastitis is one of the most common and economically significant diseases in dairy cattle, severely affecting animal health, welfare, milk yield, and quality. It remains a major challenge to sustainable dairy production worldwide [1,2].

Mastitis is the inflammation of the udder tissue, resulting from the invasion of the mammary gland by pathogens. The occurrence of mastitis is influenced by multiple factors, including environmental factors, such as milking methods, housing type, microclimate, production season, and nutrition. Udder inflammation can also result from intrinsic factors such as immune status, milk yield, age, lactation phase, stress, and udder morphology [3,4].

Mastitis is the most costly disease in European dairy farms, causing significant economic losses. It can affect up to 50% of cows during their lives, even in herds with good hygiene practices, although prevalence varies across farms [5]. Infection of a single udder quarter can decrease milk production by at least 10%. The production of safe, high-quality milk depends on udder health, which is crucial for global nutrition [6]. The clinical form of mastitis features visible inflammatory changes in milk and udder tissue, with or without systemic clinical signs. In contrast, the subclinical form does not show obvious signs of mastitis but increases somatic cell count. Cows with subclinical infections can serve as a source of infection for susceptible animals in the herd [7]. Detecting subclinical mastitis requires cow-side tests such as the California mastitis test (CMT) or laboratory tests such as somatic cell count (SCC) and milk bacteriological culture. Early diagnosis of subclinical mastitis is vital to prevent infection spread, minimize damage to udder tissue, and ensure successful treatment [8,9]. Therefore, developing rapid, accurate, and non-invasive screening methods is essential for timely diagnosis and effective control.

The development of artificial intelligence (AI) technologies, particularly computer vision, offers innovative methods for monitoring and enhancing animal welfare [10]. Simultaneously, AI has experienced significant advancements, including developments in machine learning (ML), neural networks, and deep learning (DL), which have demonstrated strong performance across multiple domains [11]. To improve operational efficiency, economic sustainability, and environmental outcomes in intensive livestock systems, smart livestock farming (SLF) is emerging as a successor to precision livestock farming (PLF) [12]. Given the rising demand for SLF, traditional animal production and management methods are now considered outdated and insufficient for addressing contemporary livestock challenges [13].

Inflammation in the udder leads to increased blood flow and metabolic activity, resulting in localized heat that can be detected by infrared thermography (IRT). The IRT is a non-invasive, remote, and passive technique that measures the surface temperature of a body through the infrared radiation of the electromagnetic spectrum that it emits. Each pixel represents a temperature value, with the coolest areas appearing blue or black and the warmest areas appearing white or red [14,15]. When combined with ML algorithms, IRT may provide a supportive screening approach for automatically identifying temperature variations associated with mastitis, while reducing reliance on manual visual observation [16].

This imaging approach is based on the principles of infrared radiation, which has a longer wavelength than visible light and a shorter wavelength than microwaves. IRT detects infrared radiation emitted from the body surface and converts it into a thermal image in which pixel intensity reflects surface-temperature variation. IRT is a rapid, non-invasive, and safe imaging approach that can detect changes in udder surface temperature associated with inflammatory reactions [17].

Researchers have established a positive relationship between udder skin surface temperature and the California mastitis test (CMT) and the Somatic Cell Count (SCC) score [14,17]. Recently, subclinical mastitis (SCM) in dairy cattle has been identified and predicted using machine-learning algorithms, which integrate and analyze data from various sources [18,19,20]. ML approaches to database analysis using digital technologies such as IRT are innovative and exciting tools for generating information for herd health monitoring and for reducing the negative effects of SCM in dairy cows [16].

Therefore, the contribution of this study lies in combining IRT with EfficientNetB3-based dual-pooling feature extraction, multiple conventional and neural-network classifiers, and leakage-aware cow-level validation. In addition to image classification, exploratory biomarker and feeding-system analyses were included to examine biological and management contexts without overstating causality. We hypothesized that IRT-derived thermal features combined with ML models could support non-invasive mastitis screening in dairy cows, although further validation is required before practical farm deployment.

Therefore, the objective of this study was to evaluate different ML models for detecting mastitis-related thermal patterns from IRT images and to explore, without causal inference, whether biomarker and feeding-system variables were associated with the observed mastitis-related outcomes.

## 2. Materials and Methods

This experiment was conducted at Alexandria Copenhagen Farm, Delta Misr Farm, and the Department of Animal and Fish Production, Faculty of Agriculture, Alexandria University. All procedures were approved and authorized by the Institutional Animal Care and Use Committee of Alexandria University (Protocol ID: Alex. Agri. 082410121). This study aimed to evaluate IRT integrated with ML models for non-invasive mastitis screening and to explore whether differences between farms and feed rations were associated with mastitis incidence. As the analysis did not identify statistically significant direct nutritional effects, the feeding system analysis was treated as exploratory.

### 2.1. Animals and Farm Description

The study was conducted on Holstein dairy cows from two dairy farms. Cows were selected randomly with varying degrees of lineage, between 10 and 260 days in lactation and average of live body weight of 620 ± 32 kg. Both farms operated under intensive management systems. Cows were housed in free-stall barns with controlled ventilation systems. Feeding was based on a total mixed ration (TMR), with differences in nutritional composition between the two farms. Image acquisition was performed under broadly stable farm conditions; however, exact ambient temperature and humidity ranges were not continuously recorded.

### 2.2. Thermal Imaging

Thermal images were captured immediately before milking using a UNI-T UTx313 thermal imaging camera. Images were acquired at an approximate distance of 1 m from the udder. The camera was positioned in approximately frontal orientation and nearly perpendicular to the right or left udder half. Before image acquisition, the udders were cleaned and dried to reduce the influence of surface moisture or contamination on thermal measurements. Images were acquired under stable farm conditions, and acquisition settings were kept consistent as far as possible. However, exact ambient temperature and humidity ranges were not continuously recorded, which is acknowledged as a limitation of the study. The key technical specifications of the UTx313 thermal imaging camera relevant to udder thermal-image acquisition are provided in Supplementary Table S1.

### 2.3. Data Loading and Preprocessing

#### 2.3.1. Chemical Analysis of TMR

Experimental diets were dried in a forced-air oven at 55 °C for 72 h, ground using a Wiley mill grinder to pass through a 1 mm stainless steel screen, and subsequently analyzed for dry matter (DM), organic matter (OM), ether extract (EE), and crude protein (CP) according to AOAC [21]. Neutral detergent fiber (NDF) and acid detergent fiber (ADF) were determined using an Ankom fiber analyzer (Fiber Analyzer A200; Ankom Technology, Macedon, NY, USA) [22]. The ingredients of the total mixed ration (TMR) and the chemical composition on a DM basis for the cows on the two farms are provided in Supplementary Table S2. Nutrient-density values were calculated using NRC [23] dairy equations. The feeding-system comparison was interpreted as exploratory because only two farms were included, and potential farm-level confounders such as management, hygiene, and environmental conditions could not be fully separated from ration effects.

#### 2.3.2. Milk Yield and Sampling

The cows were milked three times daily at 04:00, 12:00, and 20:00 in a milking parlor equipped with automatic cow identification, a milk recording system, and automated detacher milker units, which exist at Copenhagen (DeLaval herringbone) and Delta Misr (DeLaval rapid exit) farms. Individual milk yield was recorded daily using the Delaval program with the rapid exit system. Every week, individual milk samples (50 mL of milk from each cow) were collected and immediately analyzed using the California Mastitis Test (CMT). Subsequently, SCC, as well as electrical conductivity (EC) and milk composition (consisting of fat, protein, added water, solids-not-fat (SNF), freezing point, lactose, and density), were determined using a milk analyzer (Ekomilk Horizon Unlimited, Stara Zagora, Bulgaria). SCC was used as an additional comparative indicator for the biological-assessment group.

For the machine-learning image dataset, mastitis labels were assigned according to the California Mastitis Test (CMT) result at the cow/whole-udder level. CMT-negative cows were labelled as healthy, whereas CMT-positive cows were labelled as mastitic. Right- and left-udder-side thermal images obtained from the same cow were assigned the same cow-level diagnostic label. Quarter-level diagnosis was not performed, and clinical and subclinical mastitis cases were not analyzed separately.

Moreover, microbiological evaluation of coliform bacteria and *Staphylococcus aureus* was performed on milk samples collected from dairy cows in the biological-assessment group (n = 85). The colony-count technique was used, and typical black, shiny colonies surrounded by a clear halo were counted according to ISO 4832 [24] and ISO 6888-1 [25], respectively. No significant differences were observed for coliform bacteria or *Staphylococcus aureus* among samples, suggesting that these microorganisms were not the predominant detected causative agents in the examined cases. Other pathogens or non-microbial factors may have contributed to the observed mastitis-related conditions.

#### 2.3.3. Blood Sampling

Blood samples (10 mL, n = 85) were collected weekly before milking from the jugular vein of cows included in the biological-assessment group during the morning hours over five weeks. The samples were taken in clot activator tubes (Vacutainer, Becton Dickinson, Franklin Lakes, NJ, USA) and centrifuged at 3000 rpm for 20 min at room temperature. The serum was then harvested and stored at −20 °C until analysis. Biochemical parameters in the serum were determined using commercial colorimetric kits, including total protein [26], albumin [27], and glucose [28]. Globulin concentration was calculated as the difference between total protein and albumin. Beta hydroxybutyrate (BHBA) [29], non-esterified fatty acids (NEFAs) [30], lactate dehydrogenase (LDH) [31], IgG, IgA, and IgE [32] were also measured. These parameters indicate animal health, immunity, and energy balance and were used for exploratory biological contextualization of mastitis-related status rather than as definitive independent validation endpoints for the machine-learning model. Variables were selected based on their biological relevance to mastitis.

### 2.4. Mastitis Detection Using Machine Learning and Statistical Analysis

#### 2.4.1. Acquisition of Thermograms

A dataset of 976 thermal udder images was collected from Holstein dairy cows at early, mid, and late lactation stages from dairy farms in Egypt. The dataset included 708 healthy and 268 mastitic images. The images were acquired immediately before milking in the milking parlor. The 976 thermal udder images were obtained from 488 cows, with right- and left-udder-side images assigned to the same cow-level group identifier before train-test splitting. When more than one image was available from the same cow, all images from a given cow were kept within the same training or held-out test subset to reduce animal-level data leakage. Camera position, imaging distance, and acquisition settings were kept as consistent as possible during image capture.

#### 2.4.2. Data Augmentation and Balancing

To address the class imbalance while preventing data leakage, the original images were first split by cow ID into training and held-out test subsets. Data augmentation was then applied only to the training set. Images in the training subset were augmented using the Keras ImageDataGenerator until both classes were balanced at the class count. Transformations included rotation up to ±30°, width and height shifts up to 10% of image dimensions, zoom up to 20%, horizontal flipping, and nearest-neighbor filling for border pixels. The held-out test set was kept unaugmented and contained only original images.

#### 2.4.3. Dataset

The image dataset consisted of BMP-format thermal udder images from two classes, healthy and mastitic cows. The BMP images represented thermal udder-image outputs from the camera and were used consistently for image-based feature extraction; raw pixel-level radiometric temperature values were not used for model training. Images were loaded from separate directories using OpenCV (cv2), resized to 224 × 224 pixels, and processed as shown in Table 1.

**Figure 1:**
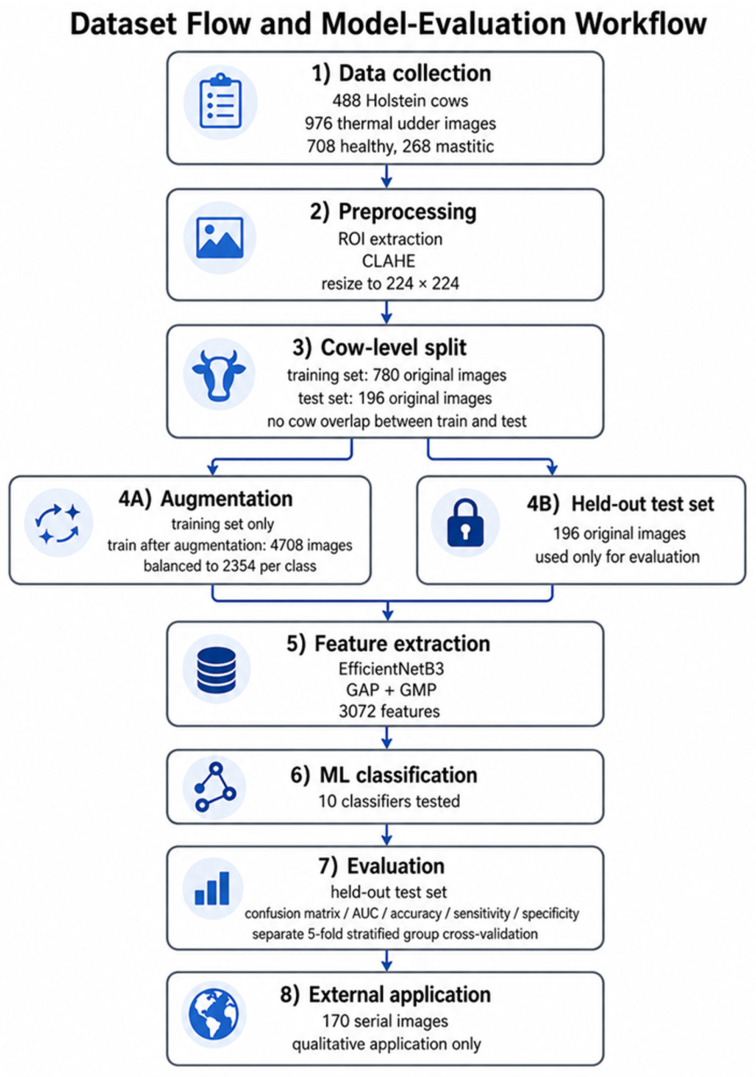
Dataset flow and model-evaluation workflow showing cow-level splitting before augmentation, training-only augmentation, held-out testing, animal-level stratified group cross-validation, and qualitative external application of the selected model.

The complete dataset flow, including cow-level splitting before augmentation, training-only augmentation, held-out testing, animal-level stratified group cross-validation, and qualitative external application, is summarized in Figure 1.

#### 2.4.4. CLAHE Contrast Enhancement

All images underwent Contrast Limited Adaptive Histogram Equalization (CLAHE) to enhance local contrast and improve microstructural visibility. It was applied exclusively to the Luminance (L) channel in the LAB color space to avoid color distortion. It is preferred over global histogram equalization as it prevents over-amplification of noise in homogeneous regions [33]. The udder-focused region of interest was used before feature extraction to reduce the influence of irrelevant background information.

#### 2.4.5. Deep Feature Extraction

##### Transfer Learning with EfficientNetB3

Feature extraction was performed using EfficientNetB3, a member of the EfficientNet family of convolutional neural networks introduced by Tan and Le. [34]. EfficientNet models employ a compound-scaling method that uniformly scales network depth, width, and resolution using a set of fixed scaling coefficients, resulting in superior accuracy–efficiency trade-offs compared to conventional architectures. EfficientNetB3 was loaded with ImageNet pre-trained weights and used as a fixed feature extractor (including top = false), without fine-tuning the convolutional weights. This transfer learning approach is justified by the limited size of the medical image dataset and the proven generalizability of ImageNet features to medical imaging tasks [35].

##### Span and Level Feature Representation

Two complementary pooling strategies were applied to the final convolutional feature maps to produce a rich feature representation:

- Level features (Global Average Pooling-GAP) computes the spatial average of each feature map channel, capturing the overall intensity distribution and global texture patterns across the entire image.
- Span features (Global Max Pooling-GMP) captures the maximum activation in each feature channel, highlighting the presence of the most discriminative local features regardless of their spatial location.

The GAP and GMP vectors were concatenated to form the final feature vector, resulting in a 3072-dimensional representation (1536 × 2) per image. This dual-pooling strategy, referred to as ‘Span and Level’ feature extraction, has been shown to outperform single-pooling approaches by capturing both global context (level) and local discriminative regions (span).

##### Feature Standardization

Following feature extraction, all feature vectors were standardized using a standard scaler (zero mean, unit variance). The scaler was fitted on the training set and applied to both training and test sets to prevent data leakage. Feature standardization is essential for distance-based and gradient-based classifiers to prevent features with larger magnitudes from dominating the learning process.

#### 2.4.6. Machine-Learning Classification

##### Train/Test Split

Before augmentation, the dataset was partitioned using GroupShuffleSplit with cow ID as the grouping variable (80% training and 20% testing; random seed = 42). This ensured that no individual cow appeared in both training and testing subsets. The resulting training subset contained 780 original images (566 healthy and 214 mastitic), and the held-out test subset contained 196 original images (142 healthy and 54 mastitic). After training-only augmentation, the training set was balanced at 2354 images per class (4708 images total).

##### Classifiers (Model Training and Evaluation)

Ten ML models (decision tree, Gaussian NB, AdaBoost, linear discriminant analysis (LDA), random forest, extra trees, logistic regression, K-nearest neighbors (KNNs), support vector machine (SVM), and multi-layer perceptron (MLP) were used and evaluated on the extracted features (Table 2).

##### Evaluation Metrics

For each classifier, predictions were evaluated on the held-out test set using the confusion matrix and standard diagnostic-performance metrics. The positive class was mastitis, whereas the negative class was healthy. The confusion matrix was used to derive true positives, true negatives, false positives, and false negatives, from which accuracy, sensitivity, specificity, precision, F1-score, and AUC were calculated.

Each classifier model’s performance was evaluated on the held-out test set using the following predictive metrics [20].

Precision: proportion of positive predictions that are truly mastitis. It measures the total number of true positives (TPs) divided by the total number of predicted positives and was calculated as:

Precision = TP/(TP + FP).

Accuracy (Acc): proportion of correctly classified samples; it measures the total number of correct classifications divided by the total number of cases and was calculated as:

Acc = (TP + TN)/(TP + TN + FP + FN).

Sensitivity (Se): also known as recall, sensitivity is the proportion of true mastitis cases correctly identified, is critical for minimizing missed diagnoses, and was calculated as:

Se = TP/(TP + FN).

Specificity (Sp): specificity is the proportion of true healthy cases correctly identified, as described in the equation:

Sp = TN/(TN + FP).

F1-Score: a single metric that is a harmonic mean of precision and recall, as described in the equation:

F1 Score = (2 × Precision × Se)/(Precision + Se).

Area Under the Receiver Operating Characteristic Curve (AUC-ROC): It measures the discriminative ability across all classification thresholds. The best-performing model was selected based on the highest AUC-ROC score, as AUC is more informative than Acc for imbalanced or threshold-sensitive medical classification tasks.

### 2.5. Statistical Analysis—Logistic Regression

#### 2.5.1. Rationale for Logistic Regression

Binary logistic regression was selected as the statistical method for investigating the relationship between continuous biomarker variables and binary mastitis outcomes (0 = healthy, 1 = mastitis). Unlike linear regression, logistic regression constrains predicted values to the 0–1 range and models the log-odds of the outcome as a linear function of the predictor, making it the standard method for binary outcome analysis in veterinary and clinical research [36].

#### 2.5.2. SCC vs. CMT and ML Prediction: Simple Logistic Regression

In the first regression analysis, somatic cell count (SCC) was used as the sole predictor variable to model two binary outcomes: (1) CMT result (positive/negative) and (2) ML model prediction (mastitis/healthy). This analysis was intended to assess whether model predictions followed the expected SCC–mastitis relationship rather than establish definitive clinical validation.

#### 2.5.3. SCC Data Cleaning

The SCC values contained non-standard string entries. Values recorded as ‘<90,000’ were replaced with the midpoint estimate of 45,000 cells/mL. Values recorded as ‘>9,000,000’ were assigned the boundary value of 9,000,000 cells/mL. All comma separators were removed before numeric conversion, and remaining non-convertible entries were treated as missing and excluded.

#### 2.5.4. Outcome Encoding

The CMT results containing the keyword ‘positive’ were binary encoded as 1 (mastitis), and all other results as 0 (healthy). ML model predictions containing ‘mastitis’ were encoded as 1 and ‘healthy’ predictions as 0. Encoding was performed using case-insensitive string matching to ensure robustness.

#### 2.5.5. Model Fitting and Visualization

L1-penalized logistic regression was fitted independently for each outcome using statsmodels for *p*-value estimation and scikit-learn LogisticRegression with the liblinear solver for plotting predicted probabilities. Predicted probabilities were computed over 300 evenly spaced SCC values spanning the observed range to generate smooth curves. *p*-values were reported as returned by the penalized logistic models without post-hoc forcing or adjustment.

#### 2.5.6. L1-Penalized Logistic Regression: Multi-Variable Analysis

For the multi-variable analysis across blood and milk parameters, L1-penalized (Lasso) logistic regression was applied using the statsmodels library. L1 regularization was chosen over L2 because it performs automatic feature selection by shrinking irrelevant coefficients to zero, which is particularly valuable when analyzing a large panel of potentially correlated biomarkers.

#### 2.5.7. Variables Analyzed

The following independent variables were analyzed against the ML model prediction outcome (Table 3).

#### 2.5.8. Handling Complete Separation

Complete separation is a common problem in logistic regression with small veterinary datasets, occurring when a predictor perfectly separates the two classes, causing maximum likelihood estimates to be infinite and standard errors to be undefined. L1 regularization was employed to constrain coefficient magnitudes and produce finite estimates when possible [37]. The regularization parameter alpha was set to 0.01 for most variables and increased to 0.1 for APP (%) and total protein, which were more prone to separation. Maximum iterations were set at 5000 to support convergence.

#### 2.5.9. Statistical Significance

Statistical significance of each predictor was assessed using the Wald test *p*-value extracted from the regularized logistic regression results when a stable *p*-value was returned. A significance threshold of *p* < 0.05 was applied. The results were reported as significant or not significant without manual *p*-value adjustment.

#### 2.5.10. Visualization

Logistic regression results were visualized using a *p*-value summary plot and a separate SCC-based logistic regression comparison between CMT and ML predictions. These visualizations were used to summarize biomarker significance and to assess whether ML predictions followed the expected SCC-related mastitis pattern.

### 2.6. Model Evaluation Strategy

#### 2.6.1. Internal Validation—Held-Out Test Set

Formal image-level model evaluation was performed on the held-out 20% cow-level test set, which included 196 original images from the total dataset of 976 images. This test set was not augmented and was not used during model training, feature scaling, parameter selection, or classifier optimization. Because the training set was augmented only after cow-level splitting, the held-out test results are interpreted as within-dataset image-level performance.

#### 2.6.2. Animal-Level Stratified Group Cross-Validation

To obtain a more conservative estimate of animal-level generalization, a separate 5-fold stratified group cross-validation was conducted using cow-level grouping. This procedure ensured that all images from the same cow were assigned exclusively to either the training or testing subset within each fold. The cross-validation results were used to evaluate the robustness of model performance under stricter animal-level separation.

#### 2.6.3. Qualitative External Application—Serial Images

The best-performing model was additionally applied to 170 serial thermal udder images as a qualitative external application. The final prediction distribution was summarized as the number and percentage of images classified as mastitis-positive or healthy. Because no independent ground-truth labels were available for these serial images, this analysis was not treated as independent external validation.

## 3. Results

### 3.1. Performance of Machine Learning Models

The analysis evaluated 10 machine-learning classifiers using thermal udder-image features extracted from EfficientNetB3. The evaluation was performed on the unaugmented cow-level held-out test set and was supplemented by animal-level stratified group cross-validation.

Table 4 summarizes the confusion-matrix counts for each classifier on the unaugmented held-out test set, which included 196 original images: 142 healthy and 54 mastitic images. The positive class was mastitis. Among the 10 classifiers, the MLP model showed the best overall image-level performance.

On the unaugmented cow-level held-out test set, the MLP achieved the highest image-level accuracy and AUC among the ten classifiers.

**Figure 2:**
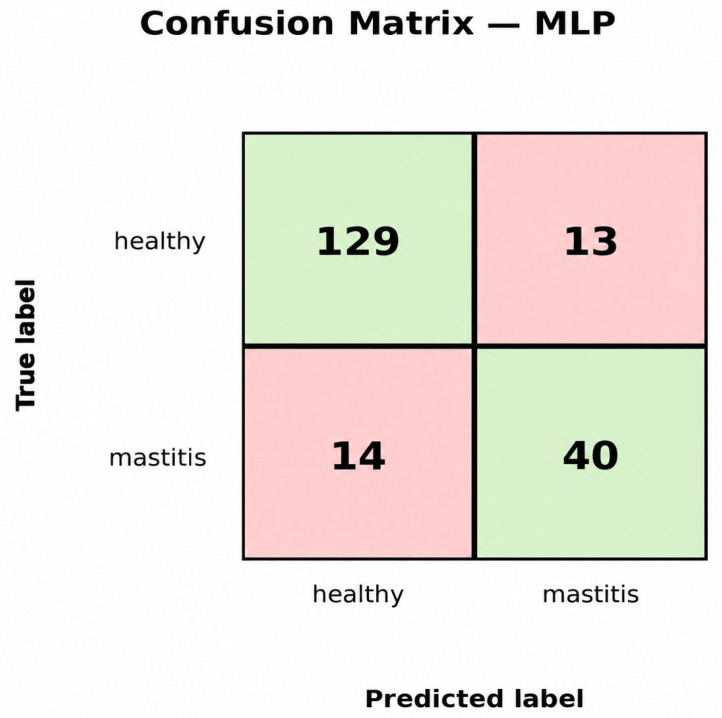
Confusion matrix of the MLP classifier in the 20% cow-level held-out test set.

Figure 2 shows the confusion matrix of the MLP classifier evaluated on the 20% held-out cow-level test dataset. The confusion matrix showed 129 true negatives, 40 true positives, 13 false positives, and 14 false negatives. For the selected MLP model, 14 mastitic images were misclassified as healthy.

Table 5 summarizes the comparative accuracy and AUC values of the ten machine-learning models on the unaugmented held-out test set. MLP achieved the highest image-level accuracy (0.8622) and AUC (0.9184; bootstrap 95% CI: 0.8740–0.9557), followed by SVM, with an accuracy of 0.8367 and AUC of 0.8963. For the selected MLP model, the bootstrap 95% confidence intervals were 0.8163–0.9082 for accuracy, 0.8740–0.9557 for AUC, 0.6250–0.8519 for sensitivity, 0.8603–0.9524 for specificity, and 0.6457–0.8320 for F1-score.

The ROC analysis confirmed that MLP and SVM were the leading classifiers, with MLP showing the highest AUC; the corresponding AUC values and bootstrap 95% confidence intervals are summarized in Table 5, while the full ROC curves are provided in Supplementary Figure S1. In the additional animal-level 5-fold stratified group cross-validation, the mean AUC was 0.6812 ± 0.1323, with mean precision of 0.6255 ± 0.0799, recall of 0.6725 ± 0.1733, F1-score of 0.6344 ± 0.0726, and accuracy of 0.6244 ± 0.0642.

**Figure 3:**
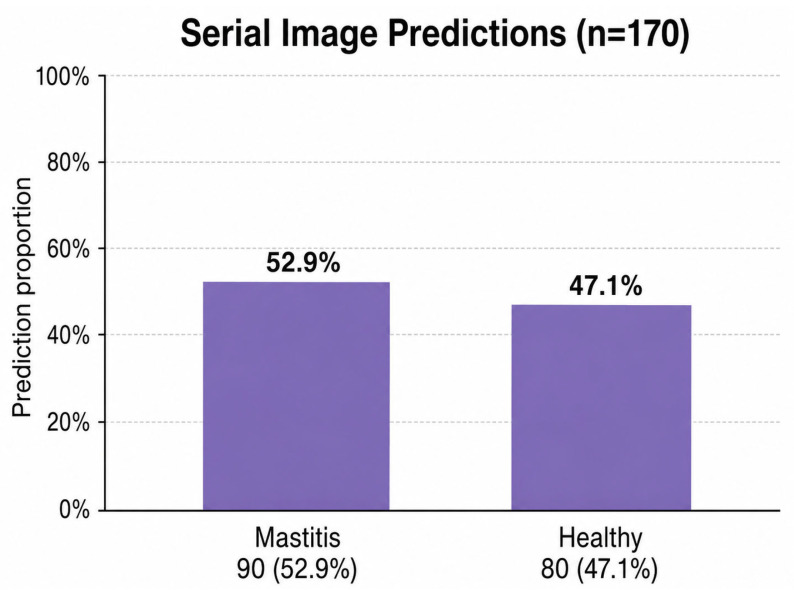
Prediction distribution for 170 serial thermal udder images used as a qualitative external application.

Since MLP was the best-performing model, it was applied to 170 serial thermal udder images as a qualitative external application. The model classified 90 images (52.9%) as mastitis-positive and 80 images (47.1%) as healthy, as shown in Figure 3. These results are presented as prediction distributions and should not be interpreted as independent external validation.

### 3.2. Exploratory Biomarker Association Analysis

**Figure 4:**
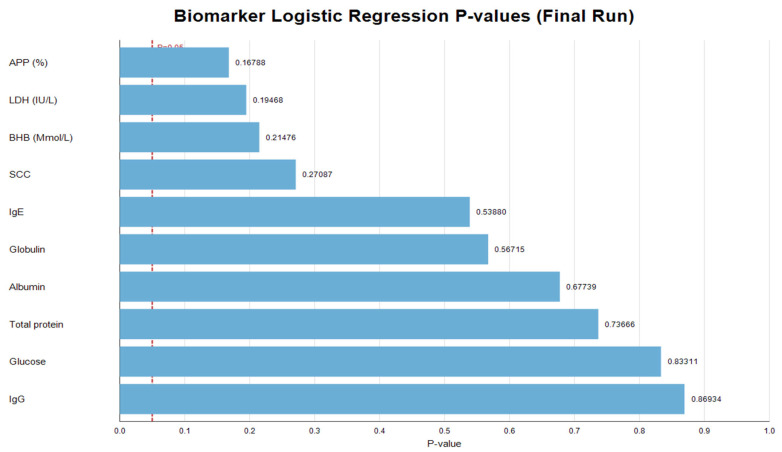
Biomarker *p*-values from L1-penalized logistic regression. No evaluated biomarker reached *p* < 0.05.

The exploratory L1-penalized logistic regression analyses did not identify statistically significant associations between the evaluated blood/milk parameters and ML-predicted mastitis status. As summarized in Figure 4 and Table 6, none of the evaluated variables reached the significance threshold of *p* < 0.05. The smallest *p*-values were observed for APP (%) (*p* = 0.16788), LDH (*p* = 0.19468), BHBA (*p* = 0.21476), and SCC (*p* = 0.27087), but none reached statistical significance. IgA and NEFA did not return stable *p*-values in the final model output and were therefore interpreted conservatively as not significant.

**Figure 5:**
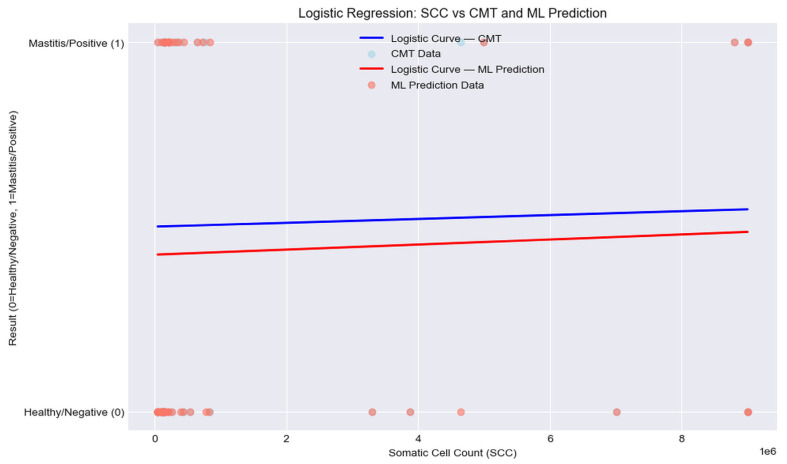
Logistic regression relationships between somatic cell count (SCC), CMT, and ML prediction. Neither CMT (*p* = 0.88908) nor ML prediction (*p* = 0.67885) showed a statistically significant SCC association.

Figure 5 compares the logistic regression relationships between SCC and the traditional California Mastitis Test (CMT) and between SCC and ML predictions. In this analysis, neither association reached statistical significance (CMT: *p* = 0.88908; ML prediction: *p* = 0.67885).

### 3.3. The Impacts of Different Dietary Factors

When the dataset was analyzed by ration/farm grouping, Ration A (Delta Misr) showed a higher observed mastitis incidence than Ration B (Copenhagen). In the final dataset, Ration A included 20 mastitic and 20 healthy cows (20/40; 50.0% incidence), whereas Ration B included 16 mastitic and 29 healthy cows (16/45; 35.6% incidence).

**Figure 6:**
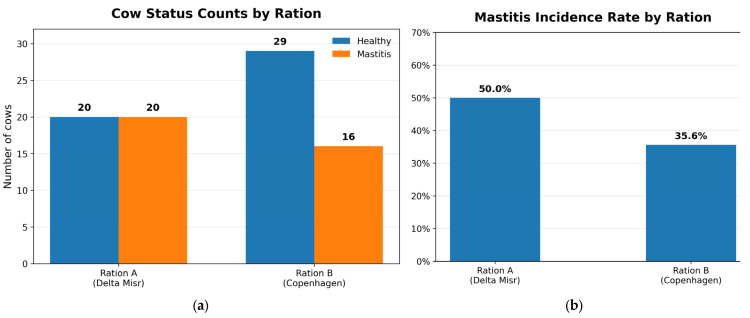
Cow status by ration/farm group: (**a**) absolute counts and (**b**) observed mastitis incidence rate.

However, logistic regression using nutritional variables did not identify a statistically significant direct nutritional effect on mastitis incidence. Crude protein (*p* = 0.18256) and TDN (*p* = 0.16665) were not significant, and NDICP, ADICP, and NEL3X did not yield stable independent *p*-values. Cow status based on the final ration/farm grouping is shown in Figure 6. The observed mastitis incidence was 50.0% for Ration A (Delta Misr) and 35.6% for Ration B (Copenhagen).

**Figure 7:**
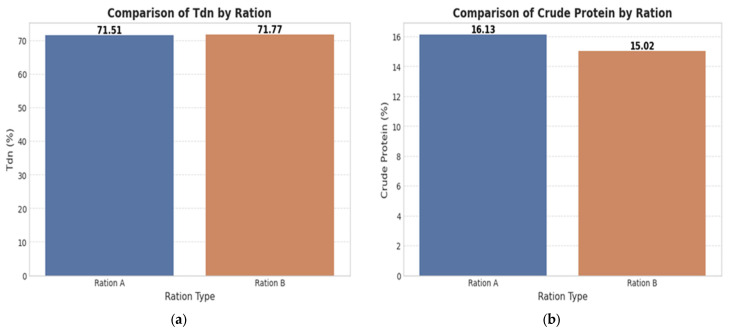
Comparison of (**a**) TDN and (**b**) crude protein with different ration types.

Figure 7 compares the crude protein (CP%) and total digestible nutrients (TDN%) between Ration A and Ration B. Ration A contained a higher crude protein content (16.13%) than Ration B (15.02%), while both rations showed nearly identical TDN values (71.51% and 71.77%, respectively), indicating similar energy availability.

**Figure 8:**
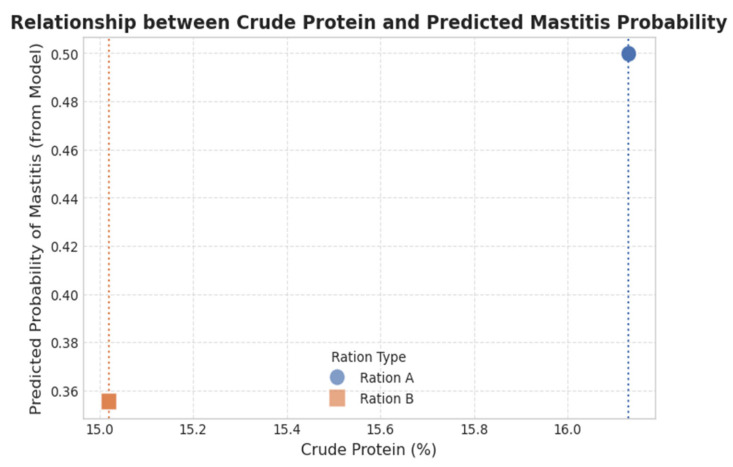
Relationship between crude protein and predicted mastitis probability.

Figure 8 illustrates the relationship between dietary crude protein content and model-predicted mastitis probability for the two ration types. The plot shows different predicted probabilities for Ration A and Ration B, but this relationship was not statistically significant in the logistic regression analysis.

The final nutritional model did not provide evidence that crude protein, TDN, or the other evaluated ration variables independently predicted mastitis status at *p* < 0.05.

## 4. Discussion

### 4.1. Interpretation of Machine-Learning Performance

The present study evaluated infrared thermography combined with EfficientNetB3-based feature extraction and conventional machine-learning classifiers for mastitis screening in dairy cows. Among the ten evaluated classifiers, the MLP model achieved the highest image-level hold-out performance, with an accuracy of 86.22% and an AUC of 0.9184. This finding suggests that nonlinear classifiers can capture discriminative thermal-image patterns associated with mastitis-related udder changes.

The superior performance of MLP may be related to its ability to model nonlinear interactions among the high-dimensional EfficientNetB3 thermal features, whereas simpler classifiers may be less able to capture complex thermal-pattern relationships associated with mastitis. However, these values should be interpreted as hold-out image-level performance rather than independent external validation. The additional animal-level 5-fold stratified group cross-validation yielded a lower mean AUC, indicating that the image-level MLP result should be considered an optimistic within-dataset estimate, whereas the group-based cross-validation provides a more conservative estimate of animal-level generalization to unseen animals.

The confusion matrix of the selected MLP model also indicates that false-negative classifications remain clinically important. For the selected MLP model, 14 mastitic images were misclassified as healthy. These false-negative cases are clinically important because missed mastitis may delay intervention and allow deterioration of udder health. Therefore, despite the relatively high specificity, the sensitivity of 74.07% indicates that the model should be considered a supportive screening tool rather than a standalone diagnostic replacement. Its potential value lies in assisting herd health monitoring and prioritizing cows for further confirmatory testing using established methods such as SCC, CMT, bacteriological culture, or molecular diagnostics.

### 4.2. Biomarker and SCC-Association Findings

The exploratory biomarker analyses did not identify statistically significant associations between the evaluated blood/milk parameters and ML-predicted mastitis status. Although SCC, immunoglobulins, acute-phase proteins, metabolic indicators, and enzyme markers are biologically relevant to mastitis and inflammatory status, the present dataset did not provide sufficient statistical evidence to confirm biomarker-model concordance. The lack of significance may reflect limited sample size, class structure, biological variability, complete-separation issues in logistic regression, or farm-level confounding.

Figure 5 compares the logistic regression relationships between SCC and the traditional California Mastitis Test (CMT) and between SCC and ML predictions. In this analysis, neither association reached statistical significance (CMT: *p* = 0.88908; ML prediction: *p* = 0.67885). Thus, the present dataset does not support the claim that ML predictions have a stronger relationship with SCC than CMT. Rather, these findings suggest that the available sample size and predictor structure were insufficient to establish robust biomarker-model concordance. Therefore, the biomarker findings should be interpreted as exploratory biological contextualization rather than confirmatory validation of the ML model.

Santana et al. [16] evaluated the use of XGBoost for diagnosing bovine subclinical mastitis from udder thermograms collected over 14 months from dairy cows in an automatic milking system. Their model incorporated thermographic, environmental, production, and animal-level variables and achieved an AUC of 0.843, with high specificity. The coldest udder-region temperature was identified as an important predictor. These findings support the potential of combining IRT with machine-learning approaches for mastitis screening, although broader validation remains necessary before routine field application.

Previous studies have also investigated mastitis screening using SCC, EC, pH, behavioral data, and thermal imaging with different machine-learning models. Tian et al. [38] implemented a KNN model with EC and pH inputs. Bobbo et al. [39] achieved an accuracy of 79.7% and a sensitivity of 52.4% using linear discriminant analysis (LDA) for mastitis diagnosis. Recently, Khan et al. [40] developed an SVM classifier based on cow behavior data. Pan et al. [41] used a machine learning-based diagnostic framework integrating logistic regression (LR), support vector machines (SVMs), and feedforward neural networks (FNNs) to evaluate mastitis detection performance with EC, SCC, and their combined inputs. The SVM model achieved the highest accuracy (95.6%) and sensitivity (100%), with SCC as the primary input, while the FNN model delivered the best overall performance with an AUC of 0.981, highlighting its ability to capture complex patterns. These results underscore the value of SCC as a reliable and specific indicator of mastitis, being less affected by non-infectious factors than EC.

Although model performance varied, these studies demonstrate that combining multiple indicators through ML-based methods offers a promising route toward more robust and accurate mastitis screening compared to single-threshold systems. Several variables may affect model accuracy, including large, high-quality annotated datasets for training, data collection under diverse climates, breeds, management practices, and human factors [42]. In agreement with this broader literature, the present study supports the potential of combining IRT with machine-learning models, while also emphasizing that broader validation remains necessary before routine field application.

### 4.3. Feeding-System Findings and Farm-Level Confounding

The feeding-system analysis showed a higher observed mastitis incidence in Ration A than in Ration B; however, the statistical analysis did not identify a significant direct nutritional predictor of mastitis status. Common indicators used to assess mastitis include SCC, EC, CMT, and milk microbiology analysis [43]. Accurate, rapid, and timely screening tools such as IRT may help characterize udder-health risk and support earlier management decisions, although pathogen identification still requires microbiological or molecular testing.

Although high dietary crude protein has been discussed in relation to metabolic load and mastitis susceptibility, the present analysis does not demonstrate a significant direct effect of crude protein on mastitis risk. Any biological interpretation must therefore remain cautious until tested in a larger dataset with balanced farm, ration, production, and management variables [44,45]. The nutritional composition of the two feeding systems may still be relevant to udder health, but the statistical analysis suggests that the observed farm differences cannot be attributed confidently to ration composition alone.

Farm-level confounders may have contributed to the observed variation, including management practices, environmental conditions, housing, hygiene, milking routine, parity, lactation stage, and sampling differences. Consequently, the feeding-system findings should be interpreted as descriptive and hypothesis-generating rather than evidence of a causal dietary effect. Future studies should use larger, prospectively balanced farm/ration designs to distinguish nutritional effects from broader management and environmental influences.

Previous research has also shown that behavioral and feeding-related indicators may contribute to mastitis monitoring. Analysis using artificial neural networks and logistic regression models has demonstrated that the time spent feeding and resting are significant behavioral indicators for mastitis detection [46]. In addition, mastitis has been associated with reduced feed intake before clinical diagnosis [47]. In this context, IRT and machine-learning approaches may complement other precision-livestock technologies by providing non-invasive thermal information related to udder health.

Recent advances in machine learning and artificial intelligence show potential for automating the inspection, detection, and analysis of thermal images and videos, supporting herd health monitoring processes [48]. IRT cameras can work synergistically with modern machine-learning models to extract thermal details and assess animal-health status [48]. Such automated systems may enable the acquisition and processing of large amounts of data, enhancing efficiency, reducing labor, and improving animal-production practices [49]. However, practical deployment should be considered a potential future application rather than an established outcome of the present pilot study.

### 4.4. Limitations and Future Work

This study has several limitations. First, mastitis labels were assigned at the cow/whole-udder level rather than at the udder-quarter level, and clinical and subclinical mastitis cases were not analyzed separately. Second, although imaging conditions were standardized as much as possible, exact ambient temperature and humidity ranges were not continuously recorded. Third, the models were trained on BMP thermal image representations rather than raw radiometric temperature matrices. Fourth, the image-level hold-out results should be interpreted as within-dataset performance, while the animal-level group cross-validation provides a more conservative estimate of generalization. Fifth, the 170 serial thermal images were used as a qualitative external application and should not be interpreted as independent external validation.

In addition, no formal model-explainability analysis, such as Grad-CAM or saliency mapping, was performed; therefore, future work should verify that model attention is concentrated on udder thermal regions rather than background or camera-related artifacts. Finally, the biomarker, bacteriological, and nutritional analyses were exploratory and may be confounded by farm-level differences in management, housing, hygiene, milking routine, and environmental conditions. Future studies should prioritize larger labeled multicenter datasets, standardized environmental recording, quarter-level diagnostic labeling, independent external validation, and prospective on-farm testing before routine practical implementation.

## 5. Conclusions

This study suggests that IRT combined with EfficientNetB3-based feature extraction and ML classification can identify mastitis-related thermal-image patterns in dairy cows within the present dataset. Among the evaluated classifiers, MLP achieved the best image-level performance, whereas animal-level group cross-validation provided a more conservative estimate of generalization. The biomarker and nutritional analyses did not identify statistically significant associations at *p* < 0.05 and should therefore be interpreted as exploratory. Overall, this work presents a transparent, leakage-aware IRT-ML pipeline for supportive mastitis screening. Larger labeled multicenter datasets and independent external validation are required before routine on-farm implementation.

## Figures and Tables

**Table 1:** Image dataset characteristics and preprocessing parameters.

Parameter	Value
Image format	BMP (Bitmap)
Input resolution	224 × 224 pixels
Color space	BGR → LAB (CLAHE on L channel) → BGR
Classes	Healthy, Mastitis
Original images	976 (708 healthy, 268 mastitis)
Training split before augmentation	780 images (566 healthy, 214 mastitis)
Held-out test split	196 original images (142 healthy, 54 mastitis)
Training set after augmentation	4708 images (2354 per class)

**Table 2:** Machine-learning classifiers and their hyperparameters.

Classifier	Type	Key Parameters
Logistic Regression	Linear	max_iter = 2000, L2 penalty
Random Forest	Ensemble (Bagging)	n_estimators = 300
Extra Trees	Ensemble (Bagging)	n_estimators = 300
AdaBoost	Ensemble (Boosting)	Default (SAMME.R)
SVM (RBF)	Kernel-based	kernel = rbf, probability = true
KNN	Instance-based	k = 7, Euclidean distance
MLP	Neural Network	layers = (256,128), max_iter = 500
Decision Tree	Tree-based	Gini impurity
Gaussian Naive Bayes	Probabilistic	Gaussian likelihood
LDA	Discriminant analysis	SVD solver

**Table 3:** Milk and blood parameters used for evaluating dairy cow health.

Variable	Unit	Category
SCC	cells/mL	Milk Quality
IgG	mg/100 mL	Immunoglobulin
IgA	mg/100 mL	Immunoglobulin
IgE	IU/mL	Immunoglobulin
APP	%	Acute Phase Protein
BHBA	mmol/L	Metabolic
NEFA	µmol/L	Metabolic
LDH	IU/L	Enzyme
Glucose	mmol/L	Metabolic
Albumin	g/dL	Protein
Globulin	g/dL	Protein
Total Protein	g/dL	Protein
Fat	%	Milk Composition
SNF	%	Milk Composition
Protein	%	Milk Composition
Lactose	%	Milk Composition
EC	mS/cm	Electrical Conductivity
Density	g/mL	Milk Physical

**Table 4:** Confusion-matrix counts of the 10 machine-learning classifiers on the unaugmented held-out test set.

Model	TP	FP	TN	FN
Logistic Regression	38	24	118	16
Random Forest	26	19	123	28
Extra Trees	27	20	122	27
AdaBoost	31	25	117	23
SVM (RBF)	41	19	123	13
KNN	35	18	124	19
MLP	40	13	129	14
Decision Tree	32	36	106	22
Gaussian NB	21	20	122	33
LDA	33	31	111	21

**Table 5:** Performance of the 10 machine-learning models on the unaugmented held-out test set.

Model	Case	Precision	Recall	F1-Score	Support	Acc	AUC	AUC 95% CI
Logistic Regression	Healthy	0.88	0.83	0.86	142	0.7959	0.8633	0.8033–0.9185
	Mastitis	0.61	0.70	0.66	54			
Random Forest	Healthy	0.81	0.87	0.84	142	0.7602	0.8303	0.7734–0.8823
	Mastitis	0.58	0.48	0.53	54			
Extra Trees	Healthy	0.82	0.86	0.84	142	0.7602	0.8483	0.7928–0.8968
	Mastitis	0.57	0.50	0.53	54			
AdaBoost	Healthy	0.84	0.82	0.83	142	0.7551	0.7784	0.7076–0.8459
	Mastitis	0.55	0.57	0.56	54			
SVM (RBF)	Healthy	0.90	0.87	0.88	142	0.8367	0.8963	0.8425–0.9408
	Mastitis	0.68	0.76	0.72	54			
KNN	Healthy	0.87	0.87	0.87	142	0.8112	0.8439	0.7798–0.9018
	Mastitis	0.66	0.65	0.65	54			
MLP	Healthy	0.90	0.91	0.91	142	0.8622	0.9184	0.8740–0.9557
	Mastitis	0.75	0.74	0.75	54			
Decision Tree	Healthy	0.83	0.75	0.79	142	0.7041	0.6695	0.5869–0.7461
	Mastitis	0.47	0.59	0.52	54			
Gaussian NB	Healthy	0.79	0.86	0.82	142	0.7296	0.8146	0.7531–0.8689
	Mastitis	0.51	0.39	0.44	54			
LDA	Healthy	0.84	0.78	0.81	142	0.7347	0.7518	0.6720–0.8253
	Mastitis	0.52	0.61	0.56	54			

**Table 6:** Statistical significance of biomarkers associated with mastitis prediction.

Biomarker	*p*-Value	Interpretation
APP	0.16788	Not significant
LDH (IU/L)	0.19468	Not significant
BHBA (mmol/L)	0.21476	Not significant
SCC (cells/mL)	0.27087	Not significant
IgE (IU/mL)	0.53880	Not significant
Globulin (g/dL)	0.56715	Not significant
Albumin (g/dL)	0.67739	Not significant
Total protein (g/dL)	0.73666	Not significant
Glucose (mmol/L)	0.83311	Not significant
IgG (mg/100 mL)	0.86934	Not significant
IgA (mg/100 mL)	N/A	Not significant; stable *p*-value not returned
NEFA (µmol/L)	N/A	Not significant; stable *p*-value not returned

## Data Availability

The original contributions presented in this study are included in the article/Supplementary Materials. Further inquiries can be directed to the corresponding author.

## Supplementary Materials

The following supporting information can be downloaded at: https://www.mdpi.com/article/10.3390/vetsci13070640/s1, Table S1: Key technical specifications of the UNI-T UTx313 thermal imaging camera relevant to udder thermal-image acquisition; Table S2: Ingredients of the TMR and chemical composition on a DM basis for lactating dairy cows in the biological-assessment group from Copenhagen and Delta Misr farms. Figure S1: ROC curves for image-level hold-out evaluation of the ten machine-learning models on the unaugmented test set. The curves represent corrected within-dataset performance and should not be interpreted as independent external validation.

## Abbreviations

IRT: infrared thermography; AI: artificial intelligence; ML: machine learning; SLF: smart livestock farming; PLF: precision livestock farming; CMT: California Mastitis Test; SCC: somatic cell count; SCM: subclinical mastitis; DM: dry matter; OM: organic matter; EE: ether extract; CP: crude protein; NDF: neutral detergent fiber; ADF: acid detergent fiber; TMR: total mixed ration; TDNs: total digestible nutrients; EC: electrical conductivity; SNF: solids-not-fat; BHBA: beta hydroxybutyrate; NEFAs: non-esterified fatty acids; LDH: lactate dehydrogenase; BMP: bitmap; CLAHE: Contrast Limited Adaptive Histogram Equalization; GAP: Global Average Pooling; GMP: Global Max Pooling; TP: true positive; TN: true negative; FP: false positive; FN: false negative; Acc: accuracy; Se: sensitivity; Sp: specificity; AUC-ROC: Area Under the Receiver Operating Characteristic Curve; AUC: Area Under the Curve; ROC: Receiver Operating Characteristic Curve; APPs: Acute Phase Proteins; LDA: linear discriminant analysis; KNN: K-nearest neighbors; SVM: support vector machine; MLP: multi-layer perceptron.

## References

[1] Velasco-Bolaños J., Ceballes-Serrano C.C., Velásquez-Mejía D., Riaño-Rojas J.C., Giraldo C.E., Carmona J.U., Ceballos-Márquez A. (2021). Application of udder surface temperature by infrared thermography for diagnosis of subclinical mastitis in Holstein cows located in tropical highlands. J. Dairy Sci..

[2] Franco-Martínez L., Muñoz-Prieto A., Contreras-Aguilar M.D., Želvytė R., Monkevičienė I., Horvatić A., Kuleš J., Mrljak V., Cerón J.J., Escribano D. (2021). Changes in saliva proteins in cows with mastitis: A proteomic approach. Res. Vet. Sci..

[3] Adkins P.R.F., Middleton J.R. (2018). Methods for diagnosing mastitis. Vet. Clin. Food Anim. Pract..

[4] Zigo F., Vasil’ M., Ondrašovičová S., Výrostková J., Bujok J., Pecka-Kielb E. (2021). Maintaining Optimal Mammary Gland Health and Prevention of Mastitis. Front. Vet. Sci..

[5] El-Sayed A., Kamel M. (2021). Bovine Mastitis Prevention and Control in the Post-Antibiotic Era. Trop. Anim. Health Prod..

[6] Coşkun G., Aytekin İ. (2021). Early Detection of mastitis by using infrared thermography in holstein-friesian dairy cows via classification and regression tree (CART) analysis. Selçuk. J. Agric. Food Sci..

[7] Swami S.V., Patil R.A., Gadekar S.D. (2017). Studies on the prevalence of subclinical mastitis in dairy animals. J. Entomol. Zool. Stud..

[8] Duarte C.M., Freitas P.P., Bexiga R. (2015). Technological advances in bovine mastitis diagnosis: An overview. J. Vet. Diagn. Investig..

[9] Lakshmi R. (2016). Bovine mastitis and its diagnosis. Int. J. Appl. Res..

[10] Araújo V.M., Rili I., Gisiger T., Gambs S., Vasseur E., Cellier M., Diallo A.B. (2025). AI-Powered Cow Detection in Complex Farm Environments. Smart Agric. Technol..

[11] Khoei T.T., Slimane H.O., Kaabouch N. (2023). Deep learning: Systematic review, models, challenges, and research directions. Neural Comput. Appl..

[12] Alshehri M. (2023). Blockchain-assisted internet of things framework in smart livestock farming. Internet Things.

[13] Kok Z.H., Mohamed Shariff A.R., Alfatni M.S.M., Khairunniza-Bejo S. (2021). Support vector machine in precision agriculture: A review. Comput. Electron. Agric..

[14] Polat B., Colak A., Cengiz M., Yanmaz L.E., Oral H., Bastan A., Kaya S., Hayirli A. (2010). Sensitivity and Specificity of Infrared Thermography in Detection of Subclinical Mastitis in Dairy Cows. J. Dairy Sci..

[15] Usamentiaga R., Venegas P., Guerediaga J., Vega L., Molleda J., Bulnes F. (2014). Infrared Thermography for Temperature Measurement and Non-Destructive Testing. Sensors.

[16] Santana R.C.M., Guimarães E.d., Caracuschanski F.D., Brassolatti L.C., Silva M.L.d., Garcia A.R., Pezzopane J.R.M., Alves T.C., Tholon P., Santos M.V.d. (2025). Machine learning techniques associated with infrared thermography to optimize the diagnosis of bovine subclinical mastitis. Vet. Med. Int..

[17] Colak A., Polat B., Okumus Z., Kaya M., Yanmaz L.E., Hayirli A. (2008). Short Communication: Early Detection of Mastitis Using Infrared Thermography in Dairy Cows. J. Dairy Sci..

[18] Lokhorst C., De Mol R.M., Kamphuis C. (2019). Invited Review: Big Data in Precision Dairy Farming. Animal.

[19] Ebrahimie E., Ebrahimi F., Ebrahimi M., Tomlinson S., Petrovski K.R. (2018). A large-scale study of indicators of subclinical mastitis in dairy cattle by attribute weighting analysis of milk composition features: Highlighting the predictive power of lactose and electrical conductivity. J. Dairy Res..

[20] Zhou X., Xu C., Wang H. (2022). The Early Prediction of Common Disorders in Dairy Cows Monitored by Automatic Systems with Machine Learning Algorithms. Animals.

[21] AOAC (2019). Official Methods of Analysis of the Association of Official Analytical Chemists: Official Methods of Analysis of AOAC International.

[22] Van Soest P.J., Robertson J.B., Lewis B.A. (1991). Methods for dietary fibre, neutral detergent fibre and nonstarch polysaccharides in relation to animal nutrition. J. Dairy Sci..

[23] NRC (2001). Nutrient Requirements of Dairy Cattle.

[24] (2006). Microbiology of Food and Animal Feeding Stuffs—Horizontal Method for the Enumeration of Coliforms—Colony-Count Technique.

[25] (2021). Microbiology of the Food Chain—Horizontal Method for Enumerating Coagulase Positive Staphylococci (*Staphylococcus aureus* and Other Species) Part 1: Method Using Baird-Parker Agar Medium.

[26] Gornall A.G., Bardawill C.J., David M.M. (1949). Determination of serum proteins by means of the biuret reaction. J. Biol. Chem..

[27] Doumas B.T., Watson W.A., Biggs H.G. (1971). Albumin standards and the measurement of serum albumin with bromcresol green. Clin. Chim. Acta.

[28] Trinder P. (1969). Determination of blood glucose using an oxidase-peroxidase system with a non-carcinogenic chromogen. J. Clin. Pathol..

[29] Taggart A.K., Kero J., Gan X., Cai T.Q., Cheng K., Ippolito M., Ren N., Kaplan R., Wu K., Wu T.J. (2005). (D)-beta-Hydroxybutyrate inhibits adipocyte lipolysis via the nicotinic acid receptor PUMA-G. J. Biol. Chem..

[30] Elshafey B.G., Elfadadny A., Metwally S., Saleh A.G., Ragab R.F., Hamada R., Mandour A.S., Hendawy A.O., Alkazmi L., Ogaly H.A. (2023). Association between biochemical parameters and ultrasonographic measurement for the assessment of hepatic lipidosis in dairy cows. Ital. Ital. J. Anim. Sci..

[31] Girdauskaitė A., Grigė S., Sabeckienė I., Džermeikaitė K., Krištolaitytė J., Miknienė Z., Antanaitis R. (2026). Associations of Blood Lactate Dehydrogenase Activity with Blood Biochemical and Automated Milk Monitoring Parameters in Early-Lactation Dairy Cows. Agriculture.

[32] Ježek J., Malovrh T., Klinkon M. (2012). Serum immunoglobulin (IgG, IgM, IgA) concentration in cows and their calves. Acta Agric. Slov. Supl..

[33] Zuiderveld K. (1994). Contrast limited adaptive histogram equalization. Graphics Gems IV.

[34] Tan M., Le Q.V. EfficientNet: Rethinking model scaling for convolutional neural networks. Proceedings of the 36th International Conference on Machine Learning (ICML).

[35] Raghu M., Zhang C., Kleinberg J., Bengio S. (2019). Transfusion: Understanding transfer learning for medical imaging. arXiv.

[36] Hosmer D.W., Lemeshow S. (2000). Applied Logistic Regression.

[37] Firth D. (1993). Bias reduction of maximum likelihood estimates. Biometrika.

[38] Tian F., Wang Z., Yu S., Xiong B., Wang S. (2020). Clinical mastitis detection by on-line measurements of milk yield, electrical conductivity and deep learn. J. Phys. Conf. Ser..

[39] Bobbo T., Biffani S., Taccioli C., Penasa M., Cassandro M. (2021). Comparison of machine learning methods to predict udder health status based on somatic cell counts in dairy cows. Sci. Rep..

[40] Khan M.F., Thorup V.M., Luo Z. (2024). Delineating mastitis cases in dairy cows: Development of an IoT-enabled intelligent decision support system for dairy farms. IEEE Trans. Ind. Inform..

[41] Pan L., Chen X., Han D., Li N., Chen D., Wang J., Chen J., Huo X. (2025). Machine learning-based clinical mastitis detection in dairy cows using milk electrical conductivity and somatic cell count. Front. Vet. Sci..

[42] Asogan A., Sazali N., Veerendra A.S., Samylingam L., Aslfattahi N., Kok C.K., Kadirgama K. (2026). A review on the impact of AI-enabled thermal imaging and IoT sensor fusion on early detection of mastitis in dairy cattle. Biosens. Bioelectron..

[43] Bobbo T., Matera R., Biffani S., Gómez M., Cimmino R., Pedota G., Neglia G. (2024). Exploring the sources of variation of electrical conductivity and total and differential somatic cell count in Italian Mediterranean buffaloes. J. Dairy Sci..

[44] Dreyer C.B., Losand H., Spiekers H., Hummel J. (2025). Influence of fat-to-protein ratio and udder health parameters on the milk urea content of dairy cows. J. Dairy Sci..

[45] Zeleke A.W., Dimonaco N.J., Lawther K., Lavery A., Ferris C., Moorby J., Huws S.A. (2025). Reducing crude protein content in the diet of lactating dairy cows improved nitrogen-use-efficiency and reduced N excretion in urine, whilst having no obvious effects on the rumen microbiome. J. Anim. Sci. Biotechnol..

[46] Grodkowski G., Szwaczkowski T., Krzysztof Koszela K., Wojciech Mueller W., Tomaszyk K., Ton Baars T., Sakowski T. (2022). Early detection of mastitis in cows using the system based on 3D motions detectors. Sci. Rep..

[47] Sepúlveda-Varas P., Proudfoot K.L., Weary D.M., von Keyserlingk M.A.G. (2016). Changes in behaviour of dairy cows with clinical mastitis. Appl. Anim. Behav. Sci..

[48] Wang M., Tan H., Li Y., Chen X., Chen D., Wang J., Chen J. (2023). Toward five-part differential of leukocytes based on electrical impedances of single cells and neural network. Cytom. Part A.

[49] Pacheco V.M., de Sousa R.V., da Silva Rodrigues A.V., de Souza Sardinha E.J., Martello L.S. (2020). Thermal Imaging Combined with Predictive Machine Learning Based Model for the Development of Thermal Stress Level Classifiers. Livest. Sci..

