# Supplementary material for: Infrared Thermography and Machine Learning for Mastitis Detection in Dairy Cows: A Pilot Case Study in Egyptian Farms
Source: Vet Sci. 2026 Jun 30;13(7):640. doi: 10.3390/vetsci13070640 (PMC13417311; doi:10.3390/vetsci13070640)

Supplementary Materials

Table S1. Key technical specifications of the UNI-T UTx313 thermal imaging camera relevant to udder thermal-image acquisition.

Parameter	Specification
Camera model	UNI-T UTx313
Detector type	UFPA, vanadium oxide
IR resolution	384 × 288 pixels
Spectral range	8–14 µm
Pixel size	12 µm
Thermal sensitivity	NETD ≤ 40 mK
Color palettes	Black hot, white hot, hottest, ironbow, rainbow

Table S2. Ingredients of the total mixed ration (TMR) and chemical composition on a dry-matter basis for lactating dairy cows in the biological-assessment group from Copenhagen and Delta Misr farms.

(a) Ingredients of the concentrate mixture, kg/head.

Copenhagen ingredient	kg/head	Delta Misr ingredient	kg/head
Corn silage	18	Corn	6.8
Alfalfa hay	6	Soya 46%	5.6
Wheat straw	0.2	Corn silage 26%	32.9
Sugar beet pellets	3	Alfalfa hay	0
Yellow corn	4.5	Wheat bran	0
Potato starch dough	2	Wheat straw	1
Tomato dregs	6.5	Rice bran	1.5
Soybean meal	1	Molasses	0.5
Bypass soy	1	Water	0
Barley dregs	6	Liquid glycerin	0.1
Flaxseed	1	Limestone	0.092
Orange peel	6	Table salt	0.055
Sodium bicarbonate	0.1	Vitamex brand new	0.022
Calci Puff	0.16	Urea	0
Ekomos	0.01	Bicarbonate	0.280
Fixphen	0.015	Magnesium oxide	0.070
Dicalcium phosphate	0.07	Selenium	0.001
Mineral	0.04	Zinc	0.005
Vitamins	0.02	Vitafix (anti-toxin)	0.1
NaCl	0.12	Dicalcium phosphate	0.025
Sulfur	0.004	Protected fat (84%)	0.465
Bioplex Manganese	0.003		
Nitro-Tech	0.019		
Niacin	0.006		
Smart Amin	0.018		
Bioplex Zinc 20%	0.005		
Selenium	0.003		
Lesygim	0.03		
Liver Tonic	0.01		
Chelated copper	0.002		
Ye-Sacc yeast	0.005		

(b) Chemical composition on a dry-matter basis.

Parameter	Copenhagen	Delta Misr
Dry matter (%)	100%	100%
Crude protein (%)	15.02	16.13
NDF (%)	30.57	30.96
ADF (%)	20.07	15.64
Cellulose (%)	17.18	13.14
Hemicellulose (%)	10.49	15.32
Lignin (%)	2.89	2.5
NDICP (%)	2.23	1.87
ADICP (%)	1.03	1.18
Crude fat (%)	4.05	3.95
Ash (%)	9.34	9.86
NFC (%)	41.02	39.11
TDN (%)	71.77	71.5087
DE1X (Mcal/kg DM) *	3.21	3.22
ME3X (Mcal/kg DM) *	2.56	2.57
ME4X (Mcal/kg DM) *	2.44	2.46
NEL3X (Mcal/kg DM) *	1.61	1.62
NEL4X (Mcal/kg DM) *	1.53	1.54

* If calcium soap of fatty acids has been included in the ration, it is not recovered in the crude fat value.

* Adjusted NDF: 28.34% on a DM basis.

* Adjusted NFC: 43.25% on a DM basis.

Note: DM, dry matter; CP, crude protein; NDF, neutral detergent fiber; ADF, acid detergent fiber; TDN, total digestible nutrients; DE, digestible energy; ME, metabolizable energy; NEL, net energy for lactation.

Figure S1. ROC curves for image-level hold-out evaluation of the ten machine-learning models on the unaugmented test set. The curves represent corrected within-dataset performance and should not be interpreted as independent external validation.

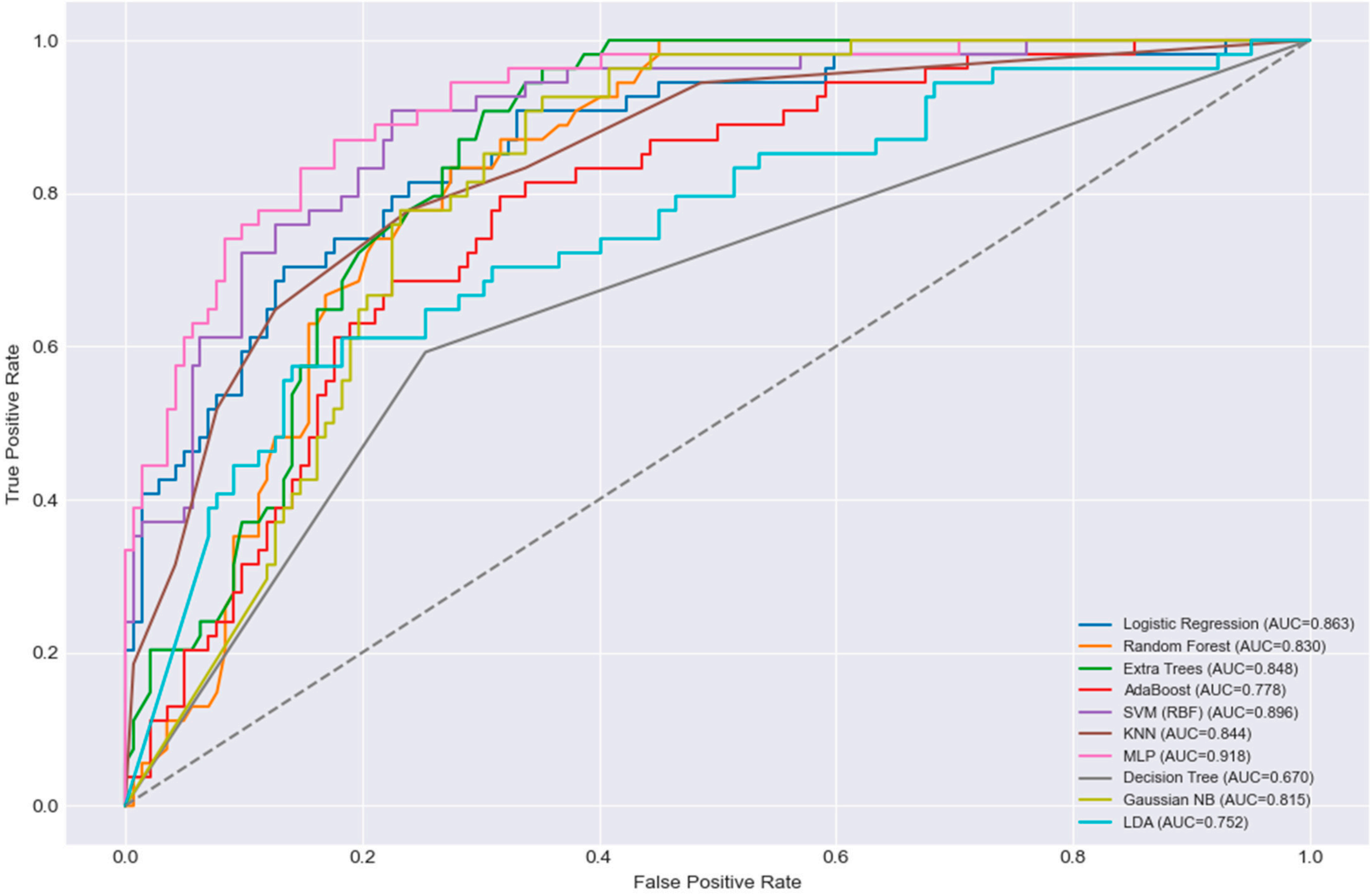

Supplement: Supplementary file 1 [file vetsci-13-00640-s001.zip › vetsci-4360533-supplementary.pdf]
